# From Comic-Con to Amazon: Fan conventions and digital platforms

**DOI:** 10.1177/14614448231165289

**Published:** 2023-05-06

**Authors:** Melanie ES Kohnen, Felan Parker, Benjamin Woo

**Affiliations:** Lewis & Clark College, USA; University of St. Michael’s College in the University of Toronto, Canada; Carleton University, Canada

**Keywords:** Comic-Con, cultural production, digital platforms, fan conventions, fandom, media industries, platformization

## Abstract

San Diego Comic-Con is North America’s premiere fan convention and a key site for mediating between media industries and fandom. In 2020, the COVID-19 pandemic forced Comic-Con to abruptly move its programming onto an array of digital platforms in an apparent “platformization” of the con. Informed by research on fan conventions, media industries, and the platformization of cultural production, this analysis of the online convention argues that Comic-Con was primed for platformization because it is already platform-like. Conventions organize markets, infrastructures, and governance to bring together attendees, media industries, and other “complementors.” Moreover, platform logics were already shaping the convention pre-pandemic in the form of experiential marketing and brand activations designed to capture attendee data. Rather than a radical break, the Comic-Con@Home online convention and in particular Amazon’s Virtual-Con activation are part of a longer process of reconfiguring the relationships between fan conventions, cultural producers, and platforms.

## Introduction

**Figure 1.**
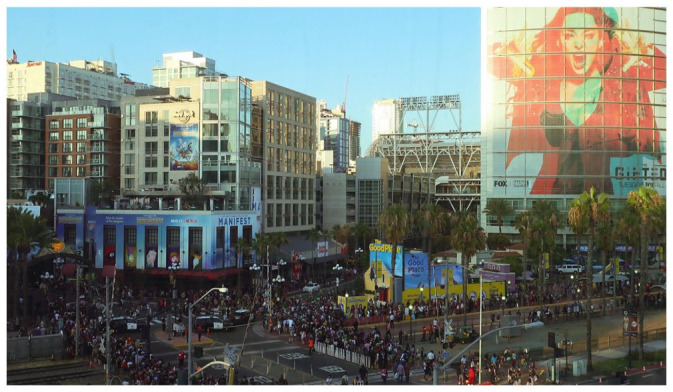
Comic-Con crowds and marketing extend into the nearby Gaslamp Quarter. Source: Photo courtesy of the authors.

Fan conventions are sites of both community-building and commerce, growing out of a century-long legacy of fans’ self-organization and institution building. In the United States, the first comic conventions were held in the 1960s so fan-collectors could meet one another, seek hard-to-find comics from dealers, and interact with creators. Despite their name, American-style comic cons have always embraced multiple media fandoms and industries (Hanna, 2020), and the best-known of these events—San Diego Comic-Con (SDCC or Comic-Con)—has especially benefited from its geographic proximity to Hollywood. From a modest 300 attendees in 1970, SDCC has grown to an estimated 130,000 attendees annually, filling the San Diego Convention Center and surrounding Gaslamp Quarter for 4 days of panels, people-watching, screenings, signings, exhibits, interactive activations, and shopping (Figure 1). Major film, television, and other entertainment media companies seeking to build buzz for new releases and in-development projects constitute a major part of this spectacle (Gilbert, 2018; Hanna, 2020; Salkowitz, 2012), but it can never be fully reduced to promotion. It is not only a space for fandom but arguably constitutes its own fan culture (Jenkins, 2012; Kohnen, 2020; Salkowitz, 2021).

Like everything else predicated on face-to-face interaction, however, fan conventions were disrupted by the COVID-19 pandemic (Woo et al., 2022). When SDCC’s organizing not-for-profit Comic-Con International (CCI) canceled the 2020 show and announced Comic-Con@Home in its place, attendees and fans were understandably unsure about what a “virtual” convention would look like. Spokesman David Glanzer promised CCI would deliver the familiar “at-show experience,” just “on the Internet” (Kornelis, 2020). To do this, Comic-Con@Home distributed the convention across a range of digital platforms, to mixed reviews (Woo et al., 2020a). Taking Comic-Con@Home as a point of departure, this article examines fan conventions through the lens of platforms and platformization. On the one hand, the emergency pivot to an online convention is a case of what we could call, following Naomi Klein (2008), disaster platformization. Confronted with the “shock” of the pandemic, under-resourced convention organizers turned to extant digital platforms to connect exhibitors, vendors, guests, and attendees; in the process, for-profit platform companies inserted themselves as new intermediaries into existing industry–con–audience networks and accelerated processes of digitization already underway. On the other hand, we argue SDCC could be spread across digital platforms in this way because comic cons *already acted like platforms*.

Our analysis emerges from past research we have individually conducted on conventions, media industries, and fandom, and an in-progress program of collaborative ethnographic research on Comic-Con. The project formally began in spring of 2020, with fieldwork originally planned for a 2021 convention that never happened. However, Comic-Con@Home prompted significant interest among our team. We worked with our collaborators to document the experience through fieldnotes, screenshots, video recordings, scraping social media hashtags, and rapid “walkthroughs” (Light et al., 2018) of various sites and platforms. The team maintained contact throughout and debriefed in online meetings following the convention. A recurring theme in these conversations was the expanding role and visibility of platforms and platform companies: Marvel/Disney and DC/Warner Bros. were largely out; YouTube and Amazon were in. But it is too simple to say that conventions were suddenly platformized in 2020. Drawing on literature on platforms and cultural production, this mixed-methods paper contextualizes these developments as one episode in a longer, more complicated story of the evolving relationships between conventions, fans, media industries, and platforms.

First, we apply a formal analysis to conventions to argue that they are a kind of platform. Thomas Poell et al. (2022: 6) remind us that not every digital enterprise is a platform; conversely, although most platform research focuses on digital media, not every platform is digital (Steinberg, 2019: 108). Cons can also be described as multisided markets that mediate between cultural producers and end-users through the provision of infrastructure and governance frameworks. To note this resemblance is not to say cons are the “original platforms.” Rather, concepts developed to analyze digital platforms can augment the growing body of fan and media industries research on conventions, how they work, and the consequences of platformization in this particular sector (Bolling and Smith, 2014; Edmunds, 2021; Gilbert, 2017, 2018; Hanna, 2020; Jenkins, 2012; Kohnen, 2014; Stanfill, 2019). Next, we turn to participant observation to document how platform logics of data collection and self-mediation are embedded in the in-person con, notably through “brand activations” mounted by film and television studios that combine experiential marketing strategies with escape rooms or immersive theater. Activations exemplify what Nicholas Carah and Sven Brodmerkel (2020: 9) call algorithmic brand culture—a cultural moment “characterised by the interplay between the open-ended and creative capacities of participants and the calculative capacities of [digital] media platforms”—and demonstrate that platformization was already reshaping SDCC before the pandemic. Finally, we return to our online ethnography of Comic-Con@Home’s use of online platforms with a particular focus on Amazon’s Virtual-Con, a self-contained online activation conspicuously organized around Amazon-owned IP and platform subsidiaries. Amazon’s “platform ecosphere” in miniature (Tiwary, 2020), Virtual-Con was a marquee sponsor of Comic-Con@Home but also sat outside the SDCC program, pointing toward a potential reconfiguration of the relationship between cons and cultural producers, and between Hollywood’s legacy entertainment industries and Silicon Valley’s tech industries (Cunningham and Craig, 2019).

For fan conventions, the pandemic was disruptive in every sense of the word, but understanding the trajectories into and out of that moment is what interests us here. Poell et al. (2022: 179) argue platformization is not a radical break but long-term transformation intensified and accelerated by the pandemic. It is precisely this mix of continuity and change that we see when we look at SDCC. SDCC’s seemingly abrupt move to digital platforms in 2020 should be viewed in the context of cons’ longer entanglement with platforms and, we contend, demonstrates that relationships between legacy media institutions and the platform economy are not simple, linear, or unidirectional. To have *any* SDCC experience during the pandemic, CCI, attendees, and cultural producers were thrown into the open arms and closed infrastructures of digital platforms, shifting focus away from Comic-Con itself and ceding power and visibility to media and tech companies. At the same time, platform companies and cultural producers learned they could promote their products, content, and services directly to online audiences, in some cases sidestepping Comic-Con entirely and gathering valuable user data in the process. Positioned at the intersection of myriad forces and interests, SDCC is a rich site for examining the complex and multiple process of platformization (Chia et al., 2020), but, in reframing both Comic-Con specifically and comic cons in general as platforms, we can see that they are not merely sites where others act but are themselves dynamic actors that structure how producers, intermediaries, and audiences interact.

## Rethinking cons and/as platforms

Social media platforms are often conceptualized as technologies that connect their users. But, as Erin Hanna (2020: 36) observes, “before platforms like Facebook, Twitter, and Tumblr existed there were letter columns, fanzines, fan clubs, and conventions,” institutions and practices that anticipated key aspects of digitally enabled participatory cultures. In this spirit, we begin by looking backwards to reconsider Comic-Con—and conventions as such (Woo et al., 2020b)—in light of recent work in platform studies. Poell et al. (2022: 5) define platforms as “data infrastructures that facilitate, aggregate, monetize, and govern interactions between end-users and content service providers.” At first glance, this definition (like many others) would seem to exclude obviously analogue conventions. At best, conventions are acted upon by platform companies. Poell et al. take the digital nature of the platforms as a given in order to focus on distinguishing platforms from other digital businesses and services that are sometimes (mis)labeled as such. After warning that “digitalization does not equal platformization,” they outline an “institutional perspective” on platforms as configurations of multisided markets, infrastructural resources, and governance strategies that insinuate themselves between cultural producers and content service providers (or “complementors”) and end-users (pp. 5–7). This markets–infrastructure–governance model provides an analytic for exploring precisely how digital platforms become indispensable intermediaries in particular industrial and geographical contexts, but neither the model itself nor the functions it seeks to explain—namely, facilitation, aggregation, monetization, and regulation—are necessarily digital. Marc Steinberg’s (2019) recent genealogy of the platform concept in Japan is useful for teasing apart the technological and non-technological features of platforms.

From inside the maelstrom of platform capitalism, Steinberg’s (2019: 108, 120) claim that “not all platforms are digital” and that the concept itself is “a-technological” may seem counterintuitive. Yet, as he demonstrates, platform didn’t become associated with computing until the mid-1980s (p. 76), and even this relatively short history has been truncated by dominant definitions in platform studies. Responding to the overwhelming academic focus on *digital* platforms, Steinberg argues that “the conditions for platform *theory* are technologically dependent, even if multisided markets are not themselves necessarily technological” (p. 104, emphasis added). In other words, the ascendancy of various kinds of digital platform businesses from the 1990s onward enabled scholars to articulate the platform concept, but that concept is not itself tethered to any particular technology. What makes credit card companies, online auction services, and ride-hailing apps platforms is not their digital infrastructure but the fact that they function as a trusted “apparatus of mediation” (p. 109).

Following Woo et al. (2020b: 21), we understand cons as events that bring people together, principally in physical spaces but with growing digital footprints, in order to circulate goods, information, discourses, and affect. Platform theory helps us make these intermediary functions visible and analyze them with more precision. Taking Poell et al.’s (2022) model as an account of transactional or mediation platforms (Steinberg, 2019: 72) sharpens “platform” into a sensitizing concept (Blumer, 1954) that is both more specific, because it is based on what platforms *do*, and broader, because it is not limited to particular (digital) industries or product categories, than definitions focused exclusively on their programmability.

### Convention markets: networking audiences and industry

**Figure 2.**
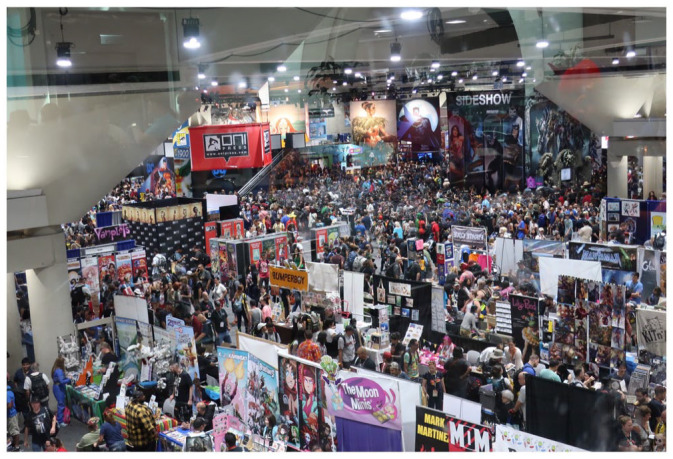
View of the exhibit floor. Source: Photo courtesy of the authors.

Acting as matchmakers between industry and audiences, SDCC and other fan conventions organize multisided markets for space, time, and attention. A multisided market is one where “platforms enable interactions between end-users, and try to get the two (or multiple) sides ‘on board’ by appropriately charging each side” (Rochet and Tirole, 2004: 2). On one side, conventions rent space from a venue and parcel it up for exhibitors and guests in the form of tables and booths on the exhibit floor or time on the programming schedule to sell things, promote things, or both. At small conventions, exhibitors might include dealers in comic books and collectibles, brick-and-mortar retail stores, and local creatives or artisans. At SDCC, they also include major entertainment companies, who set up elaborate booths, host star-studded panels, and run off-site activations (Figure 2). Warner Bros., for example, spent over $25 million annually on its promotional efforts at Comic-Con prior to the pandemic (Kit, 2022). On the other side are attendees, who typically pay at least a nominal fee for entrance. Woo et al.’s (2020b: 16) survey of 62 convention organizers found that about two-thirds charged admission fees. Large-scale events universally charge admission whether or not they are for-profit, and the costs are not trivial: a full pass to SDCC, for instance, costs $304, and badges for the planned July 2020 Comic-Con sold out within 1 hour in November 2019 (City News Service, 2019). Events may also supplement or replace this revenue stream with sponsorships, donations, or grants, potentially constituting additional sides. Importantly, multisided platform markets generate positive externalities: “when more end-users join a platform, it becomes more valuable for complementors, and vice versa” (Poell et al., 2022: 49–50). SDCC exemplifies the cross-side network effects generated by fan conventions. High attendance attracts bigger exhibitors, guests, and sponsors (complementors), while buzz about who’s exhibiting, screening new footage, selling exclusive merchandise, signing autographs, or staging activations attracts more attendees (end-users). Moreover, this virtuous circle generates attention from subcultural and mainstream entertainment journalists, reinforcing Comic-Con’s perceived importance.

### Convention infrastructures: integrating physical and digital layers

As a convention of SDCC’s scale makes clear, these events simply could not exist without a host of physical, organizational, and digital infrastructure. Physical infrastructure includes renting venues, tables, chairs, and dividers; supplying electricity; printing badges and signs; setting up stanchions to direct traffic; and so on. After SDCC 2022, attendees lamented that organizers had cut costs by foregoing the usual carpeting in the exhibit hall; sore feet from hours on concrete floors are an embodied example of how infrastructure becomes perceptible in its absence. Organizational infrastructure includes contacts, contracts, procedures, and practices that sediment organizers’ operational experience and their relationships with stakeholders and suppliers. Finally, events now make use of digital technologies to manage internal workflows, maintain data like mailing and registration lists, and conduct marketing. SDCC, for instance, uses several enterprise software platforms to manage the event space and schedule (Eventeny), badge purchases (Configio), and hotel reservations (onPeak), and it maintains a promotional presence on multiple social media platforms. However, this focus on the infrastructure that makes a con possible should not obscure the fact that SDCC and other cons are themselves infrastructure for audiences, creators, and media industries.

Cons concentrate cultural producers, intermediaries, and audiences in one place and time. This convergence is the precondition for both producers’ commercial/promotional activities and fan practices. In North America, SDCC provides unparalleled opportunities for producers to reach viewers, readers, and customers; for creators to network with potential collaborators and clients; for journalists and influencers from around the world to generate content; and for audiences to signal interest in products through in-person and digital engagement. The utility and convenience of tools provided by platforms are a key part of how cultural producers become platform dependent through “infrastructural integration” (Poell et al., 2022: 53). This conceptualization is useful for understanding how conventions structure their complementors’ actions: the “convention circuit” and “convention season” have become deeply integrated with creative professionals’ working lives; commissions and con-exclusive prints or merchandise are important supplementary revenue streams for illustrators; and professional cosplayers rely on cons as spaces for content creation and audience development. At the other end of the spectrum, although major media corporations can exert pressure on Comic-Con to accommodate their infrastructural needs or may move off-site to build their own, as discussed below, they can also be impacted by SDCC making changes to the exhibit floor, the programming schedule, or the processes by which audiences access and are regulated in these spaces.

### Convention governance: managing the flow of people, goods, and affect

The final element of Poell, Nieborg, and Duffy’s institutional platform model is governance, including the strategies of regulation, curation, and moderation. It is in this sense, rather than the infrastructural meaning (cf. Helmond, 2015), that a con is “programmable”: the selection of guests, exhibitors, and panels; the design of signage and documentation; the layout of the exhibit hall; and the management of line-ups (enforced by volunteer staff and private security) all structure what exhibitors can do and what attendees experience at the event. Programming guides, maps, souvenir programs, websites, official social media, and unofficial guides make certain aspects of the con more visible than others and steer attendees along literal and figurative pathways (Kohnen, 2020). Badges and wristbands designate different classes of attendees and the spaces they are allowed to access and what rules apply to them (or not).

**Figure 3.**
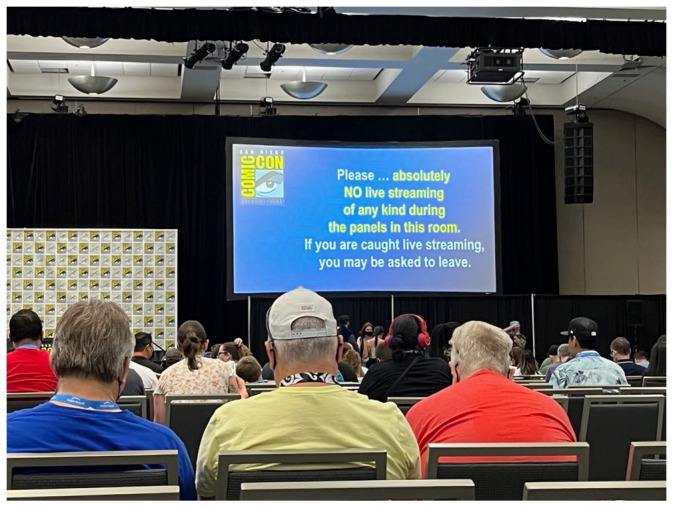
Attendees are reminded not to live stream presentations at SDCC 2022. Source: Photo courtesy of the authors.

This is not to say that there are no explicit governance frameworks. In a separate project, Woo and his students have been collecting publicly available policy documents from fan conventions’ web sites. In addition to piggybacking on venues’ existing codes of conduct, these documents usually combine regulative rules (rules that “regulate antecedently existing behaviors,” for example, *don’t* bring weapons on-site, *don’t* take someone’s photo without permission, *do* use deodorant) and constitutive ones (rules that “constitute new forms of behavior and thus regulate the very behavior that they constitute,” for example, here’s *how* you get an autograph with a guest, here’s *how* you queue to get into Hall H, this is *how* you get a refund), though with varying levels of specificity, detail, and enforcement (Searle, 2018: 52). As recently as 1991, Comic-Con’s organizers could claim they wanted “to make it possible for you to have fun, not to impose rules on you” (qtd. Hanna, 2020: 73). As of July 2022, the “Convention Policies” page on Comic-Con’s website has grown to over 2300 words in length. The longest sections relate to convention badges and to costumes, while the code of conduct is relatively parsimonious, appealing to “common sense” standards. Several policies (e.g. no running, no sitting in the aisles, and no luggage trolleys on the exhibit floor) seem designed to reduce congestion and crowding and ensure attendees’ safety. Others try to prevent people other than SDCC’s exhibitors and sponsors from taking advantage of the promotional opportunities afforded by the con (i.e. no handouts, no market research, and no retail sales outside of a purchased booth) and to ensure entertainment companies continue to proffer exclusive previews and celebrity appearances as part of the programming schedule by forbidding attendees to livestream or record panels (Figure 3). Together, these last two sets of rules govern boundaries *around* the con’s multisided market and *between* complementors and users in order to preserve the value of the platform for everyone involved and safeguard the convention’s role as platform convenor. In this sense, governance is constitutive of SDCC’s platform (Gillespie, 2018). This overview of Comic-Con’s multisided markets, infrastructural functions, and governance strategies demonstrates how the platform concept can be usefully applied to non-digital events like fan conventions. Like digital platforms, Comic-Con’s markets, infrastructures, and governance have evolved significantly in its 50-year history, in tandem with changes in the media industries and society at large. Although, as we will detail, the rise of digital platforms has had profound consequences for Comic-Con, we want to highlight cons’ own platform features. Rather than seeing platformization as a singular event imposed on cons from the outside, then, we can trace a series of *encounters* between different kinds of platforms with different goals, needs, and power dynamics, unfolding over time, with these encounters influencing Comic-Con’s mediation, digitization, and datafication.

## Hybrid platformization at SDCC

**Figure 4.**
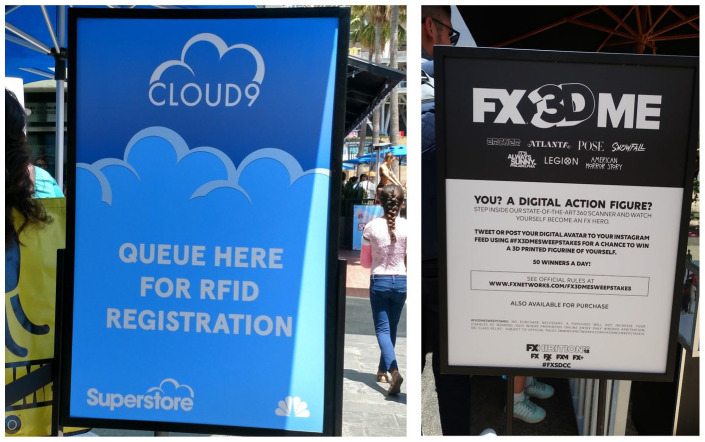
SDCC’s brand activations generate and extract user data. RFID wristband registration sign at NBC’s *Superstore* activation, 2019 (left). Sign at FX’s 2018 activation encouraging social media self-mediation (right). Source: Photo courtesy of the authors.

Film and TV studios are key complementors of SDCC’s multisided market and have long used the con as a promotional platform by hosting panels and operating booths on the show floor. These booths have increasingly integrated digital components such as sign-up forms, QR codes, RFID wristbands, and requested (or sometimes required) social media posts (Figure 4). As email addresses function as “fixed identifiers” that allow marketers to track consumers through online and offline purchases, the seemingly inconspicuous name-and-email sign-up provides valuable information to companies (Walter, 2021). Since 2010, film and TV studios have also relied on activations, including branded experiences that can take the form of escape rooms, themed restaurants, and immersive theater, to gain attendees’ attention. Located close to the convention center in downtown San Diego, activations promise an interactive deep dive into familiar storyworlds and exclusive branded give-aways, driven by a belief in experiential marketing and the experience economy, both of which prioritize in-person, affective, memorable experiences as a strategy for creating brand loyalty (Batat, 2019; Pine and Gilmore, 2011) and extend digital corporate surveillance into physical spaces (Carah and Angus, 2018; Cheney-Lipold 2017). Activations are an important component of how media companies participate in Comic-Con’s multisided market, and they rely on physical and digital infrastructures, for example, open spaces around the convention center and RFID wristbands or QR codes, for their operation. Governance takes the form of rules for lines, codes of conduct, and sometimes the requirement of an SDCC badge.

While activations incorporate key facets of platformized cultural production according to Poell et al.’s (2022) model, they also exemplify another crucial aspect of the contemporary digital platform economy, namely the central place of extracting and trading personal information. First-party data collection is a main goal of integrating various digital platforms and services into in-person activations (Kelly, 2021). As third-party data collection becomes more legally contested or banned outright, the ability to collect first-party data becomes increasingly important to film and TV companies who depend on insights into the composition of their audience (Walter, 2021). By combining the legacy aspects of SDCC’s platform, particularly the encounter of cultural producers and audiences, with digital tracking technologies, activations demonstrate how deeply entwined physical and digital spaces have become at the con and beyond. In other words, activations take on their own platform qualities: they are an enclosed space that attendees can enter without payment if they are willing to part with personal information as price of admission. In return for their attention and data, attendees can participate in an exclusive experience tailored to their pop culture interests and gain subcultural capital by sharing the experience with others on social media.

Needless to say, these transactions raise broader questions about informed consent, privacy, and commodification, given the uneven power dynamics between major media corporations and ordinary users (Andrejevic and Burdon, 2015; van Dijck, 2014). For example, at the 2019 booth for the TNT series *Snowpiercer*, attendees received a promotional pin if they followed the program’s official Instagram account. Increases in social followers, shares, and impressions are key performance indicators for activation producers (Swant, 2019). Another increasing trend at SDCC in the late 2010s was coaxing attendees into a company’s digital ecosystem, especially their video streaming services. DC/Warner Bros.’ 2019 *Pennyworth* activation asked attendees to download the Epix streaming app to gain access to a secret room (Carson, 2019), and part of the swag included a code for a free 6-month subscription to Epix Now (Nolan, 2019). This strategy results in not only the possibility of continued subscription beyond the initial 6 months, but more crucially direct access to attendees’ data and unique device IDs, sanctioned by the app’s end-user license agreement. Similarly, some activations rewarded attendees who were already members of subscription services like DC Universe or Hasbro Pulse with higher quality items than the swag given to non-members (Parks and Cons, 2019). In another increasingly common form of digital tracking, NBC’s 2019 activations required attendees to put on RFID wristbands before entering. Various stations throughout the activation required a swipe of the wristband, encoded with attendees’ personal information, in exchange for a photo op or a piece of swag, enabling granular tracking and analytics.

Stickers on the ground surrounding the 2018 activation for NBC sitcom *The Good Place* encouraged attendees to “stop and share” their experience on social media, now a near-ubiquitous feature of activations. This encouragement of “self-mediation” via social media (Khamis, 2020: 9) produces an archive ripe for algorithmic processing. On the front end of social media, attendees are tracked when they share images, videos, and industry-created hashtags; these impressions allow marketers to calculate the reach of brand messages. On the back end, this user-generated archive has the potential to train algorithms with the goal of micro-targeted advertising (Carah and Angus, 2018). The media industries perceive SDCC attendees as some of their most loyal fans, and their data can serve as a blueprint for future promotional campaigns (Kohnen, 2021). As data-driven advertising is a primary engine of the platform economy, activations function as a microcosm of that larger industry and its muddy ethical implications. Consequently, targeted data collection is one way in which activations were functioning as part of Comic-Con’s platformized ecosystem even before the pandemic.

On a broader level, the entanglement of in-person experience, mediation of said experience via social media, and algorithmic processing is not specific to activations or SDCC, but rather a defining characteristic of algorithmic brand culture. Digital platforms play a key role in algorithmic brand culture by soliciting our attention and mediating our experience, but even more importantly, by folding consumers into “fine-tuning the marketing infrastructure we become embedded within” (Carah and Brodmerkel, 2020: 8). Thus, as Carah and Brodmerkel (2020: 9) argue, “a critical theory of platforms depends on a critical theory of algorithmic brand culture,” and fan conventions are a particularly vivid instance for this theorization.

Off-site SDCC activations also point to larger cultural shifts as precursors to the stand-alone fan conventions that major media companies embraced in 2020 and beyond. After all, activations are not officially run by or part of Comic-Con even though they take place during and physically adjacent to SDCC and take advantage of its platform, massive, highly sought-after audience, and proximity to the industry hubs of “SoCal” Hollywood and “NoCal” Silicon Valley (Cunningham and Craig, 2019). In the online environment, however, it becomes harder to tell the difference between an activation at a convention and a rival event. The COVID-era proliferation of in-house branded virtual events run in parallel or totally separately from SDCC shows how the pandemic noticeably shifted the balance of power between cons, media industries, and platform companies.

## Platformizing Comic-Con@Home 2020 and Amazon Virtual-Con

About a month after stay-at-home measures to prevent the spread of COVID-19 were implemented around the world, SDCC 2020 was officially canceled on 17 April 2020, followed several weeks later by the announcement of Comic-Con@Home on 8 May. A short YouTube video with animated text and triumphant music promised “Free parking; Comfy chairs; Personalized snacks; No lines; Pets welcome; Badges for all; And a front row seat to . . . COMIC-CON@HOME” (CCI, 2020). Ahead of the event, CCI shared graphic design assets so participants could print their own Comic-Con badges and signage and recipes for approximating convention center fare. On day 1, CCI released welcome videos featuring local dignitaries, organizers, and volunteers. These gestures sought to downplay the disruption of the COVID pandemic and Comic-Con’s digital re-mediation, and even celebrate the amenities of an at-home con, efforts that did not necessarily align with what Comic-Con@Home’s virtual attendees actually experienced.

Fan conventions, as we have argued, operate in platform-like ways for industry exhibitors, attendees, and other actors, and in recent years, digital platformization has become more and more apparent in and around the con. Comic-Con@Home in some ways turned this inside out, distributing core components of Comic-Con across various digital platforms and content portals: pre-recorded panels were hosted on YouTube; the annual Masquerade costume contest and art show moved to Tumblr; a room of gaming tables became a Discord server (with some games hosted on other video conferencing platforms); screenings turned into online watch parties that required the Scener browser extension and subscriptions to Netflix, Disney+, and other streaming services; and the convention’s massive exhibit hall—one of the spaces most closely identified with SDCC as a site of visuality, spectacle, and commerce (Hanna, 2020: 127)—was replaced with an image map of the planned booth layout that linked out to exhibitors’ own online storefronts.

Rather than a centralized and organizing hub, SDCC itself became little more than a logo across these disparate components. There was relatively little opportunity for direct interaction among fans or with creators, and several significant exhibitors declined to participate, including Marvel Studios and DC Entertainment. The resulting experience was uneven. Fans on social media seemed appreciative of the effort given the extenuating circumstances, some small-scale exhibitors anecdotally reported better than average sales (Kremenek, 2020; toddland, 2020), and fan/community-led panels received significantly more viewers on YouTube than they would have in-person (thanks in part to the ability to watch them asynchronously). However, the dominant narrative in the film and television trade press was one of failure (Woo et al., 2020a). While the metrics on even the most popular SDCC 2020 videos and virtual events pale in comparison to the top content on YouTube and other platforms, presumably the flurry of con-related engagement was at least somewhat beneficial to platform companies as well. This is just one instance of the larger phenomenon of disaster platformization whereby the crisis of the pandemic allowed digital platform companies to insert themselves into virtually all aspects of society and culture (Klein, 2020).

**Figure 5.**
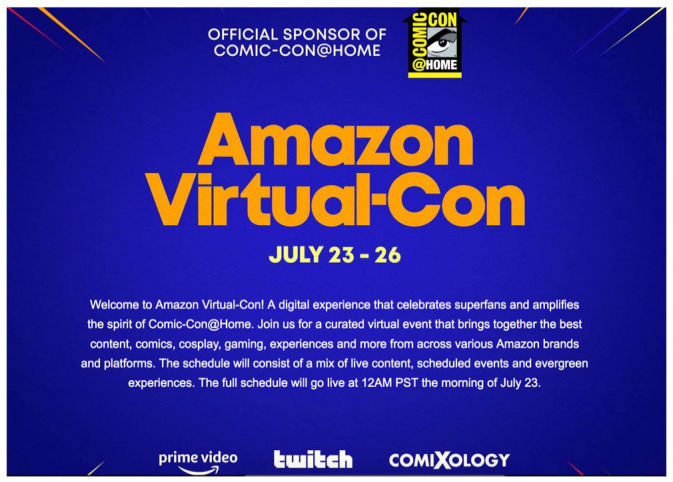
An official sponsor of Comic-Con@Home, Amazon Virtual-Con pulled people away into a separate site entirely focused on Amazon’s platform instances and IP. Source: Landing page screenshot courtesy of the authors.

The platformization of Comic-Con@Home also took other, more acute forms, however. This is most vividly illustrated by Amazon Virtual-Con, a separate-but-official “content hub” integrating and cross-promoting subsidiaries Amazon.com, Audible, ComiXology, IMDb, Prime Video, and Twitch (Amazon Studios, 2020). According to a particularly enthusiastic *Deadline* article, Virtual-Con was “a destination for fans to access and engage with Amazon’s full range of Comic-Con activations” and “gather as a community to share in the experience” (Ramos, 2020). More prosaically, it was a webpage on the Amazon.com online store with flashy comic-book inspired graphics and links to video, audio, and interactive content, divided into four categories reflecting SDCC’s main areas of cross-media, cross-fandom focus (Figure 5). Unsurprisingly, all four sections strongly emphasized platforms, services, brands, and content under the umbrella of Amazon’s corporate ownership: “SHOWS” focused on panels, interviews, and roundtables about Amazon Prime original series like *The Boys* and *Upload*, as well as IMDb TV coverage of the con and sponsored content on IGN.com; “GAMING” linked to the official @Twitch channel on Twitch.tv, where con-related programming was livestreamed throughout the event; “COMICS” included panels, demonstrations, and other content related to popular titles from a variety of publishers presented by ComiXology and Audible, some of which were also source material for shows on Prime; and “COSPLAY” featured video tutorials and demonstrations with popular cosplayers and costume designers, the majority related to Prime series including *Carnival Row* and *Good Omens*. Third-party marketing firm Digital Media Management was contracted to coordinate with the featured cosplay influencers to cross-promote their Virtual-Con videos on Instagram to “drive traffic to the virtual event” (Digital Media Management, 2020). Consistent with the broader Comic-Con@Home approach, each item listed on the main Virtual-Con page included a big yellow button entreating “attendees” to “listen now,” “learn more,” “visit channel,” or, more bluntly, “shop now,” leading to other platforms, mostly Amazon-affiliated but in some cases competitors like YouTube or Apple Podcasts.

**Figure 6.**
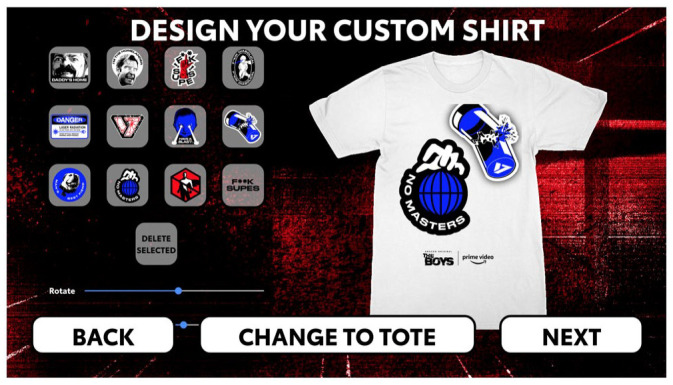
*The Boys* T-shirt design options during Amazon Virtual Con 2020. Source: Screenshot courtesy of the authors.

In addition to its broad focus on fannish/geeky media genres, Virtual-Con also adopted the rhetoric of exclusivity so commonly associated with Comic-Con (Hanna, 2020). Several interactive activities were prominently featured on the Virtual-Con hub. An elaborately designed web-based escape room promoted *Hanna*, including exclusive clips from the show as well as video recorded especially for the activation, while *The Boys* “Supes Swag” tool allowed a limited number of users to design and order customized t-shirts and other merch for free (Kohnen, 2022). Unlike much of the Virtual-Con programming, which is still online and accessible, the interactive activations were only available during Comic-Con@Home, further vaunting their exclusivity. As our collaborators Suzanne Scott and Erin Hanna observed, the “Supes Swag” promotion in particular was only intermittently available, with scarce information about how it worked (perhaps designed to keep people checking back in regularly), reminiscent of exclusive in-person con swag (Figure 6). Some fans explicitly compared it to “circling the booth” waiting for restocks at the physical con.

Much like the hybrid in-person/online instances of platformized algorithmic brand culture discussed above, various forms of user data collection characterized Virtual-Con. Much of the programming required users to have an account on platforms such as Twitch to participate in livestream chat or ComiXology to read freebie comics and previews, going beyond sponsored content to actively encourage “lock-in” to Amazon platforms and services. Interactive activities required users to sign up for mailing lists, and other forms of user data collection in exchange for access, shifting established hybrid practices from in-person activations fully online. Social media engagement was likewise encouraged, adding a layer of self-mediation to the experience and creating additional opportunities for brand engagement and data gathering. The official Twitter hashtag #amazonvirtualcon was used fairly extensively, primarily by users in select cities participating in an official prize pack giveaway of snacks, lanyards, and other branded swag. Although it is difficult to determine how successful Virtual-Con was from Amazon’s perspective, particularly since they did not reprise the event at Comic-Con@Home 2021, based on publicly visible engagement numbers it appears to have been popular.

Collectively, this menu of Amazon-branded content, its interconnected presentation on the hub page and across several platforms, and its promotional rhetoric of geeky celebration and exclusivity worked to position Virtual-Con as a self-contained con experience without ever having to leave the Amazon platform ecosystem. As Daniel Joseph (2017: 134–135) argues, although online virtual environments do not have the same limitations as physical spaces, there is still a high premium on visibility on digital platforms and storefronts. As Amazon was an official sponsor of Comic-Con@Home, links to the Virtual-Con had prominent placement on the main SDCC website and social media, and it received extensive press coverage (e.g. Al-Heeti, 2020; Darwish, 2020; Glasse, 2020; Ramos, 2020), further reinforcing the sense that it could almost stand in for the con itself, especially when many of the official SDCC panels required digging deeper into the website and often had rough, amateurish Zoom-meeting aesthetics compared to the slick professional production values of Amazon’s offerings.

Echoing the gated, increasingly elaborate Amazon off-site activations at SDCC and the staging of on-site Exhibition Hall pavilions that spatialize corporate licensing relationships (Hanna, 2020: 148–150), Virtual-Con maintained a symbolic connection to Comic-Con but constituted itself as a fully-realized branded experience of fandom and consumption that seemingly seamlessly integrated Amazon’s subsidiaries. This reflects Amazon’s broader corporate strategies, particularly around Amazon Prime Video. Ishita Tiwary’s (2020: 88) research shows that unlike competing streaming platforms, Amazon aggressively seeks to integrate users “within a portfolio of digital goods and services.” Similarly, Karen Petruska (2018: 355) argues that Amazon’s content production and distribution arms serve to “attract customers to the Amazon website to initiate, extend, and solidify a relationship between that customer and all of Amazon’s retail and service lines.” Amazon Prime Video Chief Marketing Officer Ukonwa Ojo was unambiguous about the company’s goals with activations, which she describes as “taking our customers beyond the show experience to really experience the full family of Amazon the parent brand” (Boorstin, 2022). The integrity of sub-brands, platforms, licensees, and partners like Twitch, IMDb, ComiXology, individual Prime shows and channels, and IGN are maintained (Wayne, 2018), but the design and programming of Virtual-Con clearly asserts the “larger umbrella” of its corporate brand as an overarching “platform ecosphere” built upon its underlying Amazon Web Services infrastructure, which structures much of the web (Tiwary, 2020: 88).

SDCC was the prime motivator for Virtual-Con, but SDCC itself and the geographic specificity of San Diego was largely absent for visitors to the Virtual-Con hub, its traditionally platform-like role partially subsumed by the powerful Amazon promotional engine. A preview on *Inverse* went so far as to frame Amazon as “thankfully” “stepping up” to “save” Comic-Con@Home, which otherwise “seems like a bust” due to the smaller-than-usual major media company presence, contributing to the broader media narrative of failure noted above and giving Amazon’s dominance an air of inevitability (Kleinman, 2020). In this sense, Virtual-Con is a small but notable instance of “platform capture” in which platforms enclose practices and strategies originating outside of the platform itself to their own benefit (Partin, 2020; Stanfill, 2019). As if by sleight of hand, Amazon replaced the markets, infrastructures, and governance Comic-Con had established over decades of evolution with its own, while still benefiting from SDCC’s brand recognition.

## Conclusion

SDCC is often described in excessive terms: as a kaleidoscopic spectacle, a tidal wave of humanity, impossible to communicate if you haven’t experienced it firsthand. It is indeed a massive, complexly layered event that offers different things to its many different users and publics. An a-technological, institutional conception of platforms is a vital tool for making sense of what a con like SDCC does and how it works—both before and after the rise of digital platform companies. Importantly, it helps us see connection, continuity, and entrenchment of power in the midst of disruption and change brought on by the rise of digital platforms, and to recognize conventions and their organizers as subjects as well as objects of platformization. Live events are an edge case that illuminates the broader phenomena of platformization and algorithmic brand culture. This exercise of viewing cons through a platform studies lens helps us see these processes in a new light, rather than reifying a false legacy/new media binary.

We suggest that Comic-Con was “primed” for certain kinds of platformization because it was already functioning like a platform in several important respects. In forcing SDCC online—and, specifically, onto private, for-profit digital platforms—the pandemic moment highlighted the features of cons that most resonate with platform logics. CCI essentially had no choice but to rely on these platforms in 2020 and 2021, given the time frame and resources available for their pivot to digital. In the process of this disaster platformization, however, they selected aspects of Comic-Con that were compatible with the affordances (and business models) of YouTube, Tumblr, Scener, Discord, and so on, while downplaying others, such as ephemeral moments of community among fans, which are already circumscribed by branded activations and were all but invisible during Comic-Con@Home. The spectacle of sociality and co-presence is a key draw at in-person conventions but didn’t really translate online as “participants” in an ephemeral happening became “audiences” of pre-recorded video and “users” of digital platforms. Although the online convention was potentially accessible to a much wider audience than the in-person event at San Diego, which is necessarily limited by capacity constraints at the convention center and local hotels as well as travel costs (Hanna, 2022), Comic-Con@Home provided few opportunities for its theoretically global attendees to interact with one another or with cultural producers. Moreover, in making SDCC more dependent on digital platform companies, organizers also carried along the various stakeholders—media conglomerates, licensors, creatives, dealers and vendors, professional cosplayers, and fans, collectors and other attendees—who already depended to varying degrees on SDCC as a mediation platform.

For some, these constraints also came with new opportunities. Rianka Singh (2021: 712) describes platforms as “spaces and places to amplify one’s voice, to have a speaking part in a narrative, and to display power.” Savvy cultural producers who would typically have small tables or booths at the con could leverage social media to attract more attention and direct more traffic to their online storefronts, while avoiding the travel and shipping costs ordinarily associated with exhibiting at a con. Similarly, the transformation of the convention’s programming into pre-recorded YouTube videos removed all sorts of logistical problems for attendees, who no longer had to worry about getting (and staying in) a seat for particularly popular panels or about choosing between parallel sessions; while media franchises like *The Walking Dead* still garnered the most eyeballs, the online format shifted the balance somewhat, enabling fan-led and niche panels to reach many more viewers than in a normal year.

More significantly, the sector-wide pivot to digital events demonstrated to independent and corporate cultural producers alike that they could communicate directly with fans without going through a con. Although Amazon didn’t reprise Virtual-Con for Comic-Con@Home 2021, offering instead a single Prime Video and IMDb TV–branded panel and a handful of Prime Gaming events, we see Virtual-Con as an early proof-of-concept for brand- or franchise-driven virtual events like DC FanDome, Netflix’s Tudum, and Skybound Xpo, where SDCC complementors arguably turned themselves into competitors. Although none of these events quite replicate SDCC and other cons’ full intermediary role, the promise of engaging with audiences—and collecting their data—on companies’ own terms must be compelling. These developments are consistent with wider patterns of platformization and algorithmic brand culture, in which major media companies (both legacy and Silicon Valley) continue to dominate the cultural industries despite the supposed democratization and nichification of cultural production (Poell et al., 2022: 21). At the same time, the continued centrality of the San Diego Convention Center itself, represented not only in the Comic-Con@Home virtual exhibit hall but also in third-party merchandise and a spontaneous “shrine” set up on site by local fans in 2020, suggests that the site-specific nature of cons will not be easily superseded.

**Figure 7.**
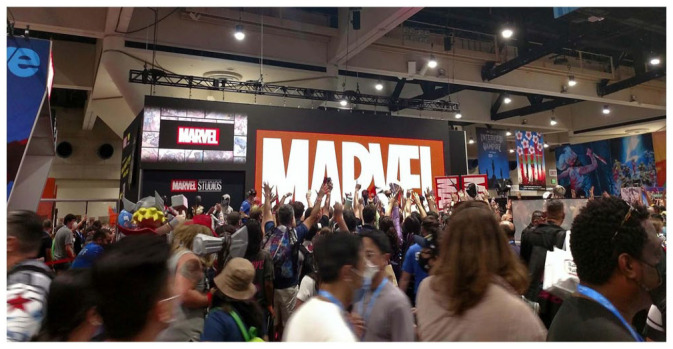
Marvel’s daily livestreams were one of the few attempts to make SDCC 2022 a hybrid event. Source: Photo courtesy of the authors.

The pandemic is not yet behind us, but conventions are back in the United States, Canada, and beyond, and the long-term impacts of this episode of disaster platformization remain uncertain. SDCC held a small-scale in-person event in November 2021 and returned to its regular dates in July 2022. The exhibit hall and Gaslamp Quarter once again featured elaborate displays and immersive activations, and Hall H and Ballroom 20 once again hosted announcements and sneak peeks from film, television, and streaming video companies (with long lines to get in). Except for Marvel’s daily livestream from their booth on the show floor and a few panels that were conducted with virtual participants (Figure 7), Comic-Con largely returned to a model of exclusive access, to the chagrin of overbooked attendees and fans who could not attend in-person (Damore, 2022; Hanna, 2020, 2022; Melden, 2022). Although this is at least partly a concession to ensure media companies’ participation in the con, it also recentered Comic-Con as a platform mediating relationships between cultural producers and fans easily accessible to Hollywood and Silicon Valley alike. Yet the framing of the returning con as “in-person” and “face-to-face” belies the continued interweaving of physical space with the digital. In algorithmic brand culture, the continuum between in-person experiences in specific spaces and social media is not incidental, but definitive: it is precisely the mediation of affect produced by platforms that generates valuable data for platforms and marketers. Consumer surveillance in the service of promotion remains a driving force for media industries.

Following Poell, Nieborg, and Duffy’s work on the platformization of cultural production, we are left with the question, are cons becoming or already platform dependent? Comic-Con@Home underscored not only how much SDCC depends on a blend of material and virtual frameworks, which increasingly includes digital platforms, but also that other actors depend on SDCC as a platform unto itself. These networks of platform dependence are key to understanding contemporary media industries and fan cultures: much like the cultural producers discussed by Poell et al., media companies, creatives, vendors, fan organizations, and individual attendees rely to varying degrees on their always-contingent access to the platform SDCC affords. SDCC’s multisided markets for time, space, and attention; physical, organizational, and digital infrastructures; and strategies governing how different entities access and make use of its spaces are what make it *the* Comic-Con and not simply *a* comic con.
